# The Utility and Safety of a Continuous Glucose Monitoring System (CGMS) in Asphyxiated Neonates during Therapeutic Hypothermia

**DOI:** 10.3390/diagnostics13183018

**Published:** 2023-09-21

**Authors:** Lucia Giordano, Alessandro Perri, Eloisa Tiberi, Annamaria Sbordone, Maria Letizia Patti, Vito D’Andrea, Giovanni Vento

**Affiliations:** 1 Department of Woman and Child Health and Public Health, Fondazione Policlinico Universitario A. Gemelli IRCCS, 00168 Rome, Italy; lucia.giordano@policlinicogemelli.it (L.G.); eloisa.tiberi@policlinicogemelli.it (E.T.); annamariasbordone@gmail.com (A.S.); marialetizia89.lp@gmail.com (M.L.P.); vito.dandrea@policlinicogemelli.it (V.D.); giovanni.vento@policlinicogemelli.it (G.V.); 2 Department of Woman and Child Health and Public Health, Catholic University of Sacred Heart, 00168 Rome, Italy

**Keywords:** continuous glucose monitoring systems, hypothermia, hypoglycemia, hyperglycemia

## Abstract

Background: The present study was designed to assess the feasibility and reliability of a Continuous Glucose Monitoring System (CGMS) in a population of asphyxiated neonates during therapeutic hypothermia. Methods: This non-randomized feasibility study was conducted in the Neonatal Intensive Care Unit (NICU) facilities of Fondazione Policlinico A. Gemelli IRCSS. Infants matching the criteria for hypothermic treatment were included in this study and were connected to the CGMS (Medtronic, Northridge, CA, USA) within the first 12 h of life. Hypoglycemia was defined as a glucose value ≤ 47 mg/dL, and hyperglycemia was defined as a glucose value ≥ 180 mg/dL. Data obtained via the CGMS were compared with those obtained via a point-of-care blood glucometer (GTX). Results: The two measuring techniques were compared using the Modified Clarke Error Grid (MCEG). Sixteen infants were enrolled. The sensor had an average (standard deviation) duration of 93 (38) h. We collected 119 pairs of glycemia values (CGMVs) from the CGMS vs. GTX measurements. The CGMS detected twenty-five episodes of hypoglycemia and three episodes of hyperglycemia. All the CGMVs indicating hyperglycemia matched with the blood sample taken via the point-of-care glucometer. Conclusions: The use of a CGMS would be useful as it could detect more episodes of disglycemia than standard care. Our data show poor results in terms of the accuracy of the CGMS in this particular setting.

## 1. Introduction

Hypoxic–ischemic encephalopathy (HIE) is an important cause of mortality and long-term neurological disabilities in term neonates. The global incidence of HIE is very variable due to the lack of a unique definition in the studies reported. In the early 2000s, HIE occurred in 1–8/1000 live term births in developed countries and in 26/1000 live births in underdeveloped countries [1]. Over the years, many improvements in prenatal and perinatal care have caused a reduction in the incidence of HIE, as reported in several European studies [2,3,4].

In high-income countries, most studies reported an incidence of HIE between 0.5 and 1.5/1000 live births, while in underdeveloped countries, a median rate of 12.1/1000 was reported [5].

Current interventions include therapeutic hypothermia [6,7,8,9], which is known to improve short-term and long-term outcomes and is the standard of care for moderate-to-severe HIE [10,11,12].

During fetal life, oxygen and glucose are delivered by an adequate cerebral blood flow to the brain. After a hypoxic insult, decreased cerebral perfusion reduces the delivery of oxygen and glucose to the brain (with a rapid depletion of glycogen stores), which leads to anaerobic metabolism, an increase in lactic acid levels.

In addition to depleted glycogen stores, infants with hypoxic–ischemic encephalopathy (HIE) may have increased peripheral glucose utilization, which can be intensified by transient hyperinsulinism.

Numerous RCTs have investigated the benefit of therapeutic hypothermia (TH) for improving the outcomes of newborns with neonatal encephalopathy (NE) [6,13,14]. The mechanisms underlying hypothermic neuroprotection are multifactorial [15]. A correlation between lower serum glucose concentrations and higher neonatal Sarnat stages was previously demonstrated [16]. In addition, initial hypoglycemia (<40 mg/dL) is an important risk factor for perinatal brain injury in neonates with HI [17]. Therefore, the strict monitoring of glucose levels is necessary for preventing and treating hypoglycemia although fluid restrictions, as indicated during hypothermic treatment, can compromise appropriate glucose delivery and may contribute to hypoglycemia. 

In neonatal hypoxic–ischemic encephalopathy, hypo- and hyperglycemia have been associated with poor outcomes (the occurrence of hyperglycemia in the first 12 h is associated with poor motor outcome [18], while hypoglycemia is correlated with the severity of encephalopathy [19]). 

Better glycemic control could avoid fluctuations in values and should be associated with a better outcome; we emphasize that this aspect is debated in the literature because there are not enough data to be able to distinguish damage due to dysglycemia in a context in which the primary disease is brain damage from asphyxia.

Newborn infants at a high risk for dysglycemia are usually monitored several times per day through finger-stick capillary blood and point-of-care (POC) blood glucometer (GTX) measurements [20]. POC measurements can result in undetected episodes of disglycemia. Tiberi et al. [21] validated the safety and reliability of the use of a continuous glucose monitoring system (CGMS) in several populations of preterm infants with no complications and good compliance from all the medical staff, improving the possibility of diagnosing disglycemia, with better therapeutic implications.

Over the years, the use of CGMSs in the pediatric population has been improved. They are currently used in the management of children with type 1 diabetes mellitus, leading to improved metabolic control in these patients. A few studies, such as Ref. [22], were conducted in preterm neonates [23,24].

A recent review shows the benefit of these glucose monitoring systems in the management of dysglycemia in the neonatal population [25]. CGMSs are new tools; at present, this technology can be considered non-inferior to standard glucose monitoring and should be introduced in neonatal settings as a supplemental tool along with traditional glucose monitoring [26]. However, the CGMS highlights a new pathway for approaching neonatal dysglycemia based on the interpretation of glycemic trends instead of punctual values of glycemia, making the diagnosis and management of this glucose disease faster and more accurate. 

The present study was designed to assess the utility and safety of a CGMS in a population of asphyxiated neonates during therapeutic hypothermia. 

## 2. Materials and Methods

This non-randomized feasibility study was conducted between 1 November 2016 and 1 July 2018 in the Neonatal Intensive Care Unit (NICU) facilities of Catholic University A. Gemelli Hospital. Infants who were admitted to the unit and matched the criteria for hypothermic treatment were included in this study. Therapeutic hypothermia was commenced (using our national guidelines) if infants ≥ 35 weeks postmenstrual age who were less than 6 h old had moderate or severe encephalopathy based on the Sarnat Grading Scale [19] and had evidence of asphyxia, which was defined by the presence of at least two of the following criteria: (1) an Apgar score < 6 at 10 min or the continued need for resuscitation with positive pressure ventilation or chest compressions at 10 min; (2) cord blood (or arterial blood sample obtained within 60 min of birth) with a pH < 7.0 mmol/L or a base deficit of 12 or more. 

Infants with major congenital abnormalities at birth or those with skin diseases were excluded. Informed consent was obtained from the parents, and the infants enrolled were connected to the CGMS (Medtronic Minimed Paradigm VEO, Northridge, CA, USA) within the first 12 h of life. Each bedside blood glucose test was prescribed by the treating physician in accordance with the standards of clinical practice during hypothermia. All GTX values detected were matched with the CGMV (CGMS’s glycemia value).

In addition, our protocols included an extra blood glucose test, to be performed with the point-of-care glucometer, when hyper- or hypoglycemia was identified via the CGMS. If the CGMS glycemia value was inaccurate (|GTX-CGMV| > 20% CGMV), an extra calibration procedure was executed for the instrument. 

The technical details concerning the CGMS used in this study were previously described in two studies by our group [21,27]. 

The CGMS comprised an ENLITE sensor, a Mini Link transmitter and a VEO monitor (Medtronic Minimed Paradigm VEO, Northridge, CA, USA). The ENLITE sensor has a cannula length of 8.75 mm, and it is equipped with glucose–oxidase which, in the presence of glucose in the interstitial space, creates an electrical current every 10 s which spreads wirelessly through a Mini Link transmitter to the VEO monitor, which estimates the average of the current measured every 5 min. The measuring principle is based on the production of hydrogen peroxide from glucose and oxygen via the enzymatic activity of glucose–oxidase. The hydrogen peroxide is then oxidized by a specific electrode that triggers the movement of electrons. The measured current is then converted into an estimate of blood glucose through the calibration procedure. The system requires calibration at least every 12 h, using the glucometer blood-glucose values. The extracts obtained are always arterialized capillaries. The interstitial glucose concentration values are expressed in mg/dL in a range between 40 mg/dL (2.2 mmol/L) and 430 mg/dL (24 mmol/L), and values that do not fall within this range are expressed as 430 mg/dL while the VEO monitor emits a sound alarm. Data are monitored and shown in real time. The insertion procedure involves the identification of an unharmed area of skin in the lateral part of the thigh and the application of a 2.5% Lidocaine–2.5% Prilocaine ointment over the area 30 min before the insertion of the ENLITE sensor. The sensor is inserted following a sterile procedure and is then connected to the MINILINK transmitter. The values documented through the continuous monitoring software were downloaded via Care Link TM after the sensor was removed.

Hypoglycemia was defined as a glucose value ≤ 47 mg/dL [28,29], and hyperglycemia was defined as a glucose value ≥ 180 mg/dL [30]. Data obtained via the CGMS were compared with those obtained via GTX, which is the most frequently used method in the NICU. 

This was a feasibility study. A formal sample size calculation was not performed as it was unnecessary. 

The two measuring techniques were compared using the Modified Clarke Error Grid (MCEG). The MCEG is a tool for investigating the accuracy of a new device for measuring glycemia in a neonatal setting and has been previously used and described [27]. We also analyzed the glucose-monitoring data. Episodes of hyper- and hypoglycemia and their mean durations were evaluated. 

A statistical analysis was executed using STATA software, v15.0.

## 3. Results

Population details are summarized in Table 1. Sixteen infants requiring therapeutic hypothermia were enrolled: twelve males and four females, with a median birth weight (BW) of 3232 g (ranging from 2895 to 3755 g) and a median gestational age of 39 weeks (range: 38–40 weeks). The sensor had an average (DS) duration of 93 (38) h. Non-adverse events were detected; the device was tolerated well by all the infants studied. We collected 119 pairs of CGMV measurements vs. GTX measurements.

**Figure 1:**
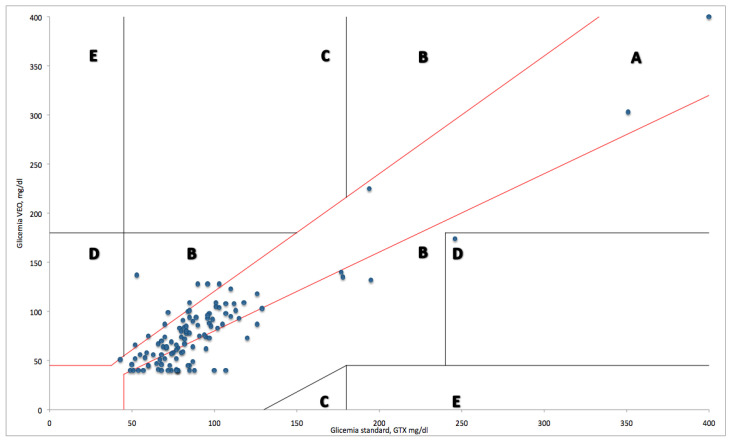
Modified Clarke Error Grid. In region A, values within 20% of the reference sensor are shown. Region B shows values which were outside 20% of the reference sensor but would not lead to an inappropriate treatment. Region C shows values leading to unnecessary treatment. Region D shows values indicating a potentially dangerous failure to detect hypo- or hyperglycemia. Region E shows values that would confuse the treatment of hypoglycemia for hyperglycemia and vice versa.

Figure 1 reports the MCEG. The MCEG shows the value generated by the monitoring system being tested along the ordinate axis, and the measurement of glucose obtained via the reference technique is shown along the abscissa axis. The MCEG identified five areas with different errors in accuracy, combined with the severity of clinical consequences. In region A, values within 20% of the reference sensor are shown. Region B shows values which were outside 20% of the reference sensor but would not lead to an inappropriate treatment. Region C shows values leading to unnecessary treatment. Region D shows values indicating a potentially dangerous failure to detect hypo- or hyperglycemia. Region E shows values that would confuse the treatment of hypoglycemia for hyperglycemia and vice versa.

In the figure below, the MCEG shows that 54.6% (65 pairs) of the measurements fall in region A, 43.7% (52 pairs) of the measurements fall in region B, and 0.84% (1 pair) of the values fall in region D. In regions C and E, no values are detected.

The CGMS detected twenty-five episodes of hypoglycemia and three episodes of hyperglycemia. Of these, only two hypoglycemic and all hyperglycemic episodes were confirmed.

In total, 54 (45%) of the 119 pairs of CGMV vs. GTX measurements were more than 20% different. Of these, in 26 (48% of 54) cases, this would lead to unnecessary treatment.

The most important factor for determining the safety of the instrument is to find the values distributed in the A and B regions. The graph shows the CGMS to be safe, but it tends to overestimate the condition of hypoglycemia, as many values in the B region are lower than 47 mg/dL in the CGMS while they are higher in the GTX evaluation. 

All the CGMVs of hyperglycemia matched with the blood sample taken via the point-of-care glucometer. Only one patient had hyperglycemia that needed treatment with insulin and was detected via the CGMS. 

All the glucose measurements achieved fell over the lower limit detected via the CGMS. 

The CGMS turned out to be safe to use in this population, and it was tolerated well after the sensor’s insertion. Moreover, it did not interfere with nursing care, and the neonatal staff showed unexpected satisfaction with respect to the CGMS.

Table 2 shows the short-term outcomes in the studied population. Eleven (69%) of the babies had a pathological brain magnetic resonance imaging (MRI) result after hypothermia, and seven (44%) individuals in the population studied had seizures.

## 4. Discussion

The CGMS offers considerable potential for the optimization of glycemia [31], and there is a growing interest in using CGM devices in neonatal intensive care. 

Previous studies have reported associations between deranged glucose homoeostasis and outcomes in infants with HIE [17,32,33,34,35].

Also, in a post hoc analysis of the CoolCap Study [36], early postnatal disglycemic profiles were associated with a greater risk of deranged multiorgan function and with the response to induced hypothermia in infants with moderate-to-severe HIE. The rate of death or severe neurological disability at 18 months of age was the lowest in normoglycemic infants. [36]

For this reason, glucose monitoring is mandatory in HIE infants. 

The use of a CGMS showing real-time glycemic values would be useful as it would lead to less invasive care and could detect more episodes of disglycemia than standard care. There are some concerns, however, about the use of these devices in HIE infants during hypothermia. 

Even if a CGMS was already used in asphyxiated neonates, this is the first time that the accuracy of a CGMS was tested in a population of term infants during hypothermia [37].

Our data show poor results in terms of the accuracy of the CGMS in this particular setting. 

The CGMS’s sensor overestimated the diagnosis of hypoglycemia, increasing the number of false positives, but it did not lead to inappropriate treatment.

New and more accurate sensors are being developed and need to be re-tested in this particular setting.

A CGMS is based on a needle sensor inserted into the subcutaneous tissue, where the device reads the glucose concentration in the interstitial fluid, which depends on the condition of the cells. Calibration procedures are considered adequate if performed under conditions of stable glucose levels [38]. However, the interstitial glucose concentration is influenced by the metabolism of the active tissues nearby, and the volume of tissue around the tip of the glucose sensor (where glucose variations in the interstitial fluid are measured via CGMS) is tiny. The active cooling of the whole body is responsible for both peripheral vasoconstriction and reduction of tissue metabolism. These processes could cause the interstitial glucose concentration to lower. Also, rapid fluctuations in the blood glucose concentration would be seen with a longer delay time than in an ordinary setting, making the CGMS’s sensors less accurate. This study has some limitations. This was a single-center feasibility study, and the population was small. Also, we did not detect a large number of hyper- and hypoglycemia episodes.

## 5. Conclusions

The CGMS tested in our study is safe but not reliable in asphyxiated neonates during hypothermic treatment, and it is tolerated well after the sensor’s insertion. 

Even if we are convinced of the CGMS’s utility in our clinical practice, our data suggest not to use the CGMS for glucose monitoring in HIE infants during a particular context such as hypothermia. Larger studies are needed to confirm these assumptions. 

The study shows that the CGMS is safe in this setting, but its accuracy needs to be improved. More data should be obtained, and a non-inferiority study is recommended to clarify the possibility of a role for the CGMS in monitoring glucose in asphyxiated neonates during hypothermia.

In the future, specific algorithms could be developed to increase the performance of the CGMS in hypothermia setting, making it possible to study and use these promising devices to better control glycemia in all the NICU’s scenarios. 

## Figures and Tables

**Table 1:** Basic population characteristics.

Sex Ratio	12/4
Gestational age (median, IQR)	39 (38–40)
Birth weight (median, IQR)	3232 (2895–3755)
Vaginal delivery (%)	62.5
Apgar score 1′ (mean, SD)	2.9 (2.48)
Apgar score 5′ (mean, SD)	4.3 (3.06)
Ph (mean, SD)	6.9 (3.55)
Base excess (mean, SD)	−18.3 (9.65)
Sarnat grade (mean, SD)	2.2 (0.96)

**Table 2:** Short-term outcomes.

	Patients
Pathological brain MRI	68.75%
Seizures	43.75%
Anticonvulsant Therapy	43.75%

## Data Availability

No new data were created or analyzed in this study. Data sharing is not applicable to this article.
